# A Dual-Payload Bispecific ADC Improved Potency and Efficacy over Single-Payload Bispecific ADCs

**DOI:** 10.3390/pharmaceutics17080967

**Published:** 2025-07-25

**Authors:** Nicole A. Wilski, Peter Haytko, Zhengxia Zha, Simin Wu, Ying Jin, Peng Chen, Chao Han, Mark L. Chiu

**Affiliations:** 1 Tavotek Biotherapeutics, 727 Norristown Road, 3 Spring House Innovation Park, Suite 101, Lower Gwynedd Township, PA 19002, USA; peter.haytko@tavotek.com (P.H.);; 2 Tavotek Biotherapeutics, Building B, LifeBay Phase 1, 999 Yinshanhu Road, Guoxiang Street, Wuzhong District, Suzhou 215000, China; zhengxia.zha@tavotek.com (Z.Z.); simin.wu@tavotek.com (S.W.); karen.jin@tavotek.com (Y.J.);

**Keywords:** ADC, dual-payload, bispecific, antibody, senescence, cancer, resistance

## Abstract

**Background/Objectives:** All current FDA-approved antibody–drug conjugates (ADCs) are single-target and single-payload molecules that have limited efficacy in patients due to drug resistance. Therefore, our goal was to generate a novel ADC that was less susceptible to single points of resistance to reduce the likelihood of patient relapse. **Methods:** We developed a dual-targeting, dual-payload ADC by conjugating a bispecific EGFR x cMET antibody to two payloads (MMAF and SN38) that had separate mechanisms of action using a novel tri-functional linker. This dual-payload ADC was tested for potency and efficacy in dividing and nondividing in vitro cell models using multiple tumor cell types. Efficacy of the dual-payload ADC was confirmed using in vivo models. **Results:** Our ADC with dual MMAF and SN38 payloads was more efficacious in inhibiting cell proliferation than single-payload ADCs across multiple cancer cell lines. In addition, the dual-payload molecule inhibited nondividing cells, which were more resistant to traditional ADC payloads. The dual-payload ADC also exhibited more potent tumor growth inhibition in vivo compared to that of single-payload ADCs. **Conclusions:** Overall, the bispecific antibody conjugated with both the MMAF and SN38 payloads inhibited tumor growth more strongly than ADCs conjugated with MMAF or SN38 alone. Developing dual-payload ADCs could limit the impact of acquired resistance in patients as well as lower the effective dose of each payload.

## 1. Introduction

Antibody–drug conjugates (ADCs) are a class of cancer therapy developed to direct drugs to protein targets that are more highly expressed on tumor cells. ADCs often use chemotherapy drugs or other toxins with the goal of limiting side effects of the drug while broadening the therapeutic index. To date, 13 ADCs have received FDA approval and are used across multiple solid and liquid tumor types [1]. While these ADCs target a variety of proteins, the payloads are mostly DNA replication inhibitors, tubulin inhibitors, and topoisomerase inhibitors. Additionally, each ADC is constructed with a single target and a single payload. While these ADCs have been successful in the clinic, patients often develop resistance [2,3,4]. Typically, mutations in the tumor cells lead to drug resistance, increased drug efflux, or target downregulation [4]. However, another mechanism leading to resistance to a broad range of cancer therapies like chemotherapy, radiation, and ADCs is the development of dormant and nondividing tumor cell populations [5,6,7,8]. This is particularly important for ADCs as the DNA replication inhibitors and tubulin inhibitors often used in ADCs are less effective in nondividing cells [5].

Tumor cells can become nondividing, or dormant, in response to chemotherapy or radiotherapy [6,8]. Migratory cancer cells can also establish dormancy for prolonged periods of time in a metastatic niche [9,10]. Dormancy is a broad term encompassing multiple nondividing cell states including senescence, which is when cells stop dividing but can secrete factors such as inflammatory cytokines, inflammatory chemokines, growth factors, and proteases that can promote tumor cell survival, tumor cell migration, and angiogenesis [11]. When considering drug-induced dormancy, senescent populations are of particular interest because of their ability to increase tumor aggression [12,13]. We hypothesize that engineering ADCs to target nondividing cancer cell populations would reduce the rates of patient relapse.

To reduce susceptibility to individual points of resistance that occured when treating with single-target, single-payload ADCs, we engineered a dual-target, dual-payload ADC using payloads with distinct mechanisms of action. While there are other dual-payload ADCs in preclinical development [14,15,16,17,18,19,20,21,22,23], many of them use two payloads from the same class of drugs [16,17,18], which do not address relapse occurring from drug resistance. Additionally, almost all dual-payload ADCs in preclinical development have an antibody toward a single target, making the ADCs susceptible to resistance by target downregulation [15,16,17,18,19,20,21,22,23]. We selected a bispecific EGFR x cMET antibody due to the combination having the following characteristics: targeting a wider range of solid tumor cell lines with overexpression of both receptors, facilitating the internalization of payloads, and being clinically validated.

For payloads, we selected MMAF and SN38, because they have distinct mechanisms of action and are clinically validated. SN38 was initially chosen due to concerns over dual payload tolerability given Dxd was shown to be 10 times more potent clinically [24]. However, both SN38 and Dxd were evaluated in vitro as single-payload ADCs with similar potency and efficacy. The non-diffusible payload MMAF was also chosen instead of the diffusible MMAE payload to complement diffusible SN38, even though MMAE has been used more often in ADCs. Pairing diffusible and non-diffusible payloads could leverage the bystander killing of diffusible payloads with the reliability of non-diffusible payloads, which were less affected by drug efflux pumps [25]. Overall, the dual-targeting, dual-payload ADC was specifically designed to be less susceptible to tumor cell resistance mechanisms.

We focused on resistance models based on nondividing and slow cycling cells which were naturally found in tumors and were often enriched after chemotherapy treatment [8]. Such cell phenotypes were more resistant to ADC-mediated killing due to increased anti-apoptotic protein expression, increased drug efflux pump activity, and reduced acidification of the lysosome among other mechanisms [26,27]. This novel dual-target, dual-payload ADC demonstrated better anti-tumor responses than the paired single-payload ADCs in multiple dividing and nondividing solid tumor models. We showed how this ADC format could better address clinically relevant resistance mechanisms.

## 2. Materials and Methods

### 2.1. Cell Lines and Generating Nondividing Cells

ADCs were tested in a panel of cell lines consisting of gastric, pancreatic, triple negative breast, and small cell lung cancers. MKN-45 (Accegen, Fairfield, NJ, USA, ABC-TC0687), BxPC-3 (ATCC, Manassas, VA, USA, CRL-1687), NCI-H196 (ATCC, Manassas, VA, USA, CRL-5823), AsPC-1 (ATCC, Manassas, VA, USA, CRL-1682), and HCC70 (ATCC, Manassas, VA, USA, CRL-2315) were cultured in RPMI 1640 medium (Corning, Corning, NY, USA, 10-041-CM) with 10% (*v*/*v*) FBS (Cytiva, Marlborough, MA, USA SH30088.03) and 1% (*w*/*v*) Penicillin-Streptomycin Solution (Corning, Corning, NY, USA, 30-002-CI). NCI-H1048 (ATCC, Manassas, VA, USA, CRL-5853) cells were cultured in Advanced DMEM/F-12 (Gibco, Waltham, MA, USA, 12634-010) with 10% FBS (Cytiva, Marlborough, MA, USA, SH30088.03) and 1% (*w*/*v*) penicillin-streptomycin. Capan-2 cells (ATCC, Manassas, VA, USA, HTB-80) were cultured in McCoy’s 5a Medium (Gibco, Waltham, MA, USA 16600-082) with 10% (*v*/*v*) FBS and 1% (*w*/*v*) penicillin-streptomycin. BT-20 cells (ATCC, Manassas, VA, USA, HTB-19) were cultured in MEM (Corning, Corning, NY, USA, 10-010-CV) with 10% (*v*/*v*) FBS and 1% (*w*/*v*) penicillin-streptomycin. Hs578t cells (ATCC, Manassas, VA, USA, HTB-126) were cultured DMEM + Glutamax (Gibco, Waltham, MA, USA, 10566-016) with 10% (*v*/*v*) FBS and 1% (*w*/*v*) penicillin-streptomycin. All cultured cells were kept in an incubator at 37 °C and 5% CO_2_.

Doxorubicin (HY-15142), gemcitabine (HY-17026), ribociclib (HY-15777), and binimetinib (HY-15202) were purchased from MedChemExpress (Monmouth Junction, NJ, USA), and stock solutions were made following by diluting the drugs to 10 mM in DMSO. To generate nondividing populations, BxPC-3 and Capan-2 cells were treated with doxorubicin (1 × 10^−7^ to 1 × 10^−10^ M), gemcitabine (1 × 10^−7^ to 1 × 10^−10^ M), or the combination of ribociclib (1 μM) and binimetinib (5 μM) for 6–7 days. Nondividing cells were then further analyzed for SA-β-gal expression to ensure populations were senescent.

### 2.2. Generation, Production, and Characterization of ADCs

The 412a and 111 antibody molecules were expressed in a stable CHO cell line grown in FortiCHO media (Thermo Scientific, Waltham, MA, USA, A1148301) at 37 °C, 8% CO_2_. Cells were separated from culture media using centrifugation. The media was applied to a MabSelect SuRre column (Cytiva, Marlborough, MA, USA, 11003493) and purified using standard Protein A techniques. The purified antibodies were buffer exchanged into PBS (Thermo Scientific, Waltham, MA, USA, 14190) using a G-25 desalting column (Cytiva, Marlborough, MA, USA, 17508702). They were evaluated using size-exclusion chromatography (Tosoh TSKgel G3000SWXL, King of Prussia, PA, USA, 0008541) and polyacrylamide gel electrophoresis (NuPAGE 4–12% Thermo Scientific, Waltham, MA, USA, NP0321 with MOPS buffer Thermo Scientific, Waltham, MA, USA, NP0001).

The proprietary trifunctional linker terminating in maleimide, propargyl, and ketone groups as synthesized by Synovel Laboratory (West Haven, CT, USA). The final dual-payload molecule was assembled following the synthetic route shown (Supplementary Figure S1). The topoisomerase inhibitor SN38 was attached via copper-catalyzed azide-alkyne cycloaddition reaction (CuAAc) with a cleavable PEG8-Val-Cit-PABC linker (Broadpharm, San Diego, CA, USA, BP-29793). The tubulin polymerization inhibitor MMAF was attached via oxime ligation with a non-cleavable PEG4-aminooxy linker (Creative Biolabs, Shirley, NY, USA). The final product was isolated by reversed phase chromatography, and its mass was confirmed to match the theoretical mass of the desired dual-payload compound using mass spectroscopy. The choice of using payloads with different mechanisms of action paired with distinct linkers reduces the impact of resistance to a single drug class, leverages the bystander killing effect of a diffusible payload like SN38 when released via a cleavable linker, and facilitates the cytosolic accumulation of a non-diffusible payload such as MMAF when released via a non-cleavable linker. MC-MMAF and CL2A-SN38 single-payload linkers were purchased from MedChemExpress (Monmouth Junction, NJ, USA).

To create the ADCs, 412a or 111 antibody molecules were bound to protein-G agarose beads using standard practice. The bound antibody was treated with an amount of 3,3′,3′′-Phosphanetriyltripropanoic acid (TCEP, Thermo Scientific, Waltham, MA, USA, 77720) sufficient to reduce the desired number of disulfide bonds to facilitate conjugation. Maleimide functionalized dual or single-payload molecules were added in excess to ensure consistent drug to antibody ratio. Excess reductant and linker-payloads were washed from the agarose beads using PBS, pH 7.2 without calcium or magnesium (Thermo Scientific, Waltham, MA, USA, 14190), and the conjugated antibody was eluted using glycine·HCl [100 mM], pH 2.7 (Teknova, Hollister, CA, USA, G4527). The pH was adjusted to 7 with 1 M Na·HEPES, pH 8.5 (Thermo Scientific, Waltham, MA, USA, J61360). The ADCs were exchanged into PBS using Amicon Ultra Centrifugal Filter tubes (Millipore Sigma, Burlington, MA, USA, UFC9010) ADCs were evaluated for drug to antibody ratio using hydrophobic interaction chromatography (HIC). The optimized HIC method was performed with a TSKgel-Butyl-NPR column (4.6 × 100 mm, 2.5 µm, TOSOH Separations, King of Prussia, PA, USA). Mobile phase A was deionized water, mobile phase B consisted of sodium phosphate [25 mM], ammonium sulfate [2 M], pH 6.5 in water; mobile phase C consisted of sodium phosphate [25 mM], isopropanol [10% *v*/*v*], pH 6.5 in water. The column temperature was maintained at 30 °C, and the flow rate was 0.33 mL/min with a gradient from A [25%], B [75%] to C [100%] over 100 min. Protein was detected by measuring absorbance at 214 ± 4 nm. Each peak was assigned a drug to antibody ratio (DAR) value based on expected values for cysteine conjugation. Average DAR was calculated using the formula Σ (peak area × DAR)/(total peak area).

### 2.3. Senescence-Associated Beta-Galactosidase Staining

To establish senescent cell models, 5 × 10^4^ Capan-2 and BxPC-3 cells were plated in cell culture media in 24 well tissue culture plates (Thermo Fisher Scientific, Waltham, MA, USA, FB012929) and placed in an incubator overnight at 37 °C and 5% CO_2_. Capan-2 and BxPC-3 cells were then treated with a range of concentrations of doxorubicin and gemcitabine from 1 × 10^−10^ to 1 × 10^−7^ M to test the optimal dosing for generating senescent cells. Capan-2 and BxPC-3 cells were also treated with set concentrations of 1 μM ribociclib (CDK4/6i) and 5 μM binimetinib (MEKi) [28]. Doxorubicin, gemcitabine, ribociclib, and binimetinib were all diluted in media from their 10 mM stock solutions. Drug-treated and untreated cells were placed in an incubator at 37 °C and 5% CO_2_ for 6–7 days before staining for senescence-associated beta-galactosidase (SA-β-gal) using a SA-β-gal Staining Kit (Cell Signaling Technologies, Danvers, MA, USA, 9860) according to the manufacturer’s instructions. Four representative images were captured per well at 20X magnification. For each image, the SA-β-gal staining area (blue colorimetric staining) and total cell area were quantified using ImageJ (FIJI, v1.54p) [29]. The percentage of SA-β-gal staining for each image was calculated using the formula SA-β-gal area/total cell area × 100. The percent SA-β-gal of each image was then averaged to generate a single data point per treatment group.

### 2.4. Cell Viability Assays

For cell viability assays using dividing cells, cells were plated at a concentration of 1 × 10^3^ cells/25 μL media in a white 384 well plate (Corning, Corning, NY, USA, 353963) and rested overnight. The next day, each antibody or ADC was serially diluted from 6 × 10^−6^ M to 6 × 10^−11^ M using a dilution factor of 1:10. The test articles were then added to the cells to create a final concentration of 1 × 10^−6^ M to 1 × 10^−11^ M. All cells besides Capan-2 cells were then placed in an incubator at 37 °C and 5% CO_2_ for 72 h. Due to slow cell division, Capan-2 cells had an incubation time of 6 days. After the designated incubation period, CellTiter-Glo 2.0 Assay (Promega, Madison, WI, USA, G9243) reagent was added to wells according to manufacturer’s instructions. Luminescence was read using a SpectraMax i3x (Molecular Devices, San Jose, CA, USA) and measured in relative light units (RLU). Luminescence data was graphed using Graphpad Prism (v10.4.1) and a nonlinear regression analysis was performed using a four-parameter logistic model to determine the IC_50_ value of each test article. The IC_50_ values were used as a readout for ADC potency. To determine efficacy of the ADCs, the readout from the highest concentration of ADC was compared to the untreated group using the formula: (untreated cells—ADC treated cells)/untreated cells × 100.

To generate nondividing Capan-2 populations, cells were plated in a white 384 well plate (Corning, Corning, NY, USA, 353963) at a concentration of 1 × 10^3^ cells/25 μL media and rested overnight in an incubator at 37 °C and 5% CO_2_. The next day, cells were treated with 100 nM doxorubicin. After incubation for 7 days, dividing Capan-2 cells were plated as described above and rested overnight. The following day, dividing and nondividing Capan-2 cells were all treated with control antibody or ADC as described above.

To generate nondividing BxPC-3s, a confluent flask of cells was treated with 1 μM CDK4/6i and 5 μM MEKi and placed in an incubator at 37 °C for 6–7 days to establish nondividing populations. After the incubation period, 2 × 10^3^ nondividing cells and 1 × 10^3^ dividing cells were plated in a white 384 well plate (Corning, Corning, NY, USA, 353963) at a concentration of 1 × 10^3^ cells/25 μL media and placed in an incubator overnight at 37 °C and 5% CO_2._ Dividing and nondividing cells were then treated with ADC or control antibody and analyzed as described above.

### 2.5. Incucyte Assays

Dividing and nondividing cells generated for cell viability assays were placed in an Incucyte S3 (Sartorius, Göttingen, Germany) at 37 °C and 5% CO_2_ after treatment with ADC or control antibody to determine if nondividing cell populations were arrested throughout our experiments. Cells were imaged at 10X every 12 h. Cell confluence of the untreated dividing and nondividing populations were graphed over time to determine if cells remained nondividing over the course of the experiment or the kinetics at which they regrew.

### 2.6. Flow Cytometry

Capan-2 cells and BxPC-3 cells were plated in a 24 well plate at 7.5 × 10^4^ Capan-2 cells/well and 5 × 10^4^ BxPC-3 cells/well and placed in an incubator overnight at 37 °C and 5% CO_2_. The next day, Capan-2 cells were treated with 100 nM doxorubicin and BxPC-2 cells were treated with 1 µM CDK4/6i and 5 µm MEKi. After 7 days, 1 × 10^5^ dividing and nondividing cells were seeded into a 96 well V-bottom plate (Corning, Tewksbury, MA, USA, 3357). A four-point dilution series of 412a and 111 antibodies were generated using 1:5 dilutions starting at 10 µg/mL. Cells were washed with FACS Stain Buffer with BSA (BD Biosciences, Franklin Lakes, NJ, USA, 554657) and incubated with each dilution of 412a or 111 antibodies for 90 min on ice. Cells were washed twice with FACS Stain Buffer with BSA (BD Biosciences, Franklin Lakes, NJ, USA, 554657) and incubated with a 1:250 dilution of Alexa Fluor 647-conjugated F(ab’)2 Fragment Goat Anti-Human IgG (Jackson Immunoresearch, West Grove, PA, USA, 109-606098) secondary antibody for 30 min on ice. Untreated and secondary only controls were also included in the experiment. Cells were washed twice and analyzed on an Attune NxT flow cytometer (Thermo Fisher Scientific, Waltham, MA, USA). Using FlowJo (v10.9.0), the bulk cell population was gated first using FSC-A x SSC-A parameters, then on single cells using FSC-A x FSC-H parameters. Mean fluorescence intensity of 412a versus 111 in the single cell dividing and nondividing populations at each concentration was assessed using FlowJo (v10.9.0).

### 2.7. In Vivo Tumor Growth Inhibition

Mice were housed in a specific pathogen-free (SPF) facility in individual ventilated cages (5 mice/cage) on corn cob bedding with crumpled tissue paper for enrichment. The SPF facility maintained a 12:12 light cycle (22 ± 1 °C, 60% humidity). Animals had ad libitum access to irradiated chow and autoclaved water. Health was monitored daily; no abnormalities were observed. Balb/c Nude mice, female, 6 to 8 weeks old (GemPharmatech Co., Ltd., Nanjing, China), were injected subcutaneously in the right flank with 1 ×10^7^ BxPC3 cells in 200 µL of a 1:1 ratio of Matrigel (Corning, Corning, NY, USA, 354234) to PBS. Upon reaching a mean tumor volume of about 150 mm^3^, a total of 30 tumor-bearing Balb/c Nude mice were randomly allocated into six groups (*n* = 4–5 per group) using a computer-generated random number sequence (Excel RAND function) and dosed with drug. The first day of dosing was set as day 0. All the tested ADCs and the isotype control were administered via intraperitoneal (IP) injection at a dose of 1, 3 or 9 mg/kg twice per week for 2 weeks. The dose levels were selected based on prior in-house dose–response studies showing efficacy and tolerability. The primary outcome was tumor volume reduction. Tumor dimensions were regularly assessed using calipers to measure the length and width. Tumor volume was calculated by 1/2 × length × width^2^. For humane endpoints, mice were euthanized if body weight loss >20% or tumor volume exceeded 3000 mm^3^. Daily monitoring included weight, tumor size, and activity. No severe adverse events (e.g., >20% weight loss) were observed in any group during the study. All procedures involving animal care, handling, and treatment complied with the guidelines of the Institutional Animal Care and Use Committee (IACUC) of Suzhou Charles River Accelerator and Development Lab (Approved Protocols: P202302160002-20240711 and P202302160002-20241219).

### 2.8. In Vivo Pharmacokinetics

Mice were housed in a SPF facility in individual ventilated cages (5 mice/cage) on corn cob bedding with crumpled tissue paper for enrichment. The SPF facility maintained a 12:12 light cycle (22 ± 1 °C, 60% humidity). Animals had ad libitum access to irradiated chow and autoclaved water. Health was monitored daily; no abnormalities were observed. 412a-SN38+MMAF ADC was administered IP into non-tumor-bearing female Balb/c nude mice at a single dose of 3 mg/kg. Serum samples were collected in one group (*n* = 3) at 1, 24, 72, and 168 h post-injection. Serum samples were collected from a second group (*n* = 3) at 6, 48, 120 and 240 h. All procedures involving animal care, handling, and treatment complied with the guidelines of the Institutional Animal Care and Use Committee (IACUC) of Suzhou Charles River Accelerator and Development Lab (Approved Protocols: P202302160002-20240711 and P202302160002-20241219).

Serum concentrations of 412a-MMAF+SN38 were quantified using a commercially available Human IgG Precoated ELISA Kit (Dakewe, Shenzhen, China, 1128162). Kit components were brought to room temperature before use. Serum samples from the 1–72 h timepoints were diluted with serum from untreated mice and standard samples were diluted to 25 µg/mL with untreated mouse serum. All standards and samples were further diluted 1:50 with 1% Bovine Serum Albumin (BSA) and an 8-point standard curve was generated starting at 500 ng/mL. After dilutions, 100 μL of each sample and standard were added to a pre-coated plate and incubated for 1 h. Plates were washed according to manufacturer’s instructions. Anti-human IgG secondary antibody was diluted at 1:1000 and 100 μL was added to each well before. The plate was incubated for 1 h and washed according to the manufacturer’s instructions, then 100 μL of TMB chromogenic reagent was added to each well. The plate was incubated in the dark and 50 µL of stop solution was added once blue color developed. Optical density was measured at 450 nm.

Pharmacokinetic (PK) analysis was performed using a composite sampling scheme at each timepoint for an average of 3 mice.

### 2.9. Statistical Analyses

All data in which statistics are shown were tested for normality using a Shapiro–Wilk test before performing additional statistical analyses. If the Shapiro–Wilk test returned a *p*-value greater than 0.05, the data were considered to be normally distributed and a one-way ANOVA was performed. If the Shapiro–Wilk test returned a *p*-value less than or equal to 0.05, the data were considered to be non-normally distributed and a Kruskal–Wallis test was performed. Statistics were generated using Graphpad Prism (v10.4.1). Tumor doubling time was assessed by modeling tumor volume as a function of time using R (v4.4.0) and RStudio (v1.4). PK modeling was performed using ADAPT 5 (v5.0.65) to determine the half-life and Cmax values using a one-compartment model.

## 3. Results

### 3.1. A Novel Tri-Functional Linker Was Generated to Accommodate MMAF and SN38 Payloads

**Figure 1:**
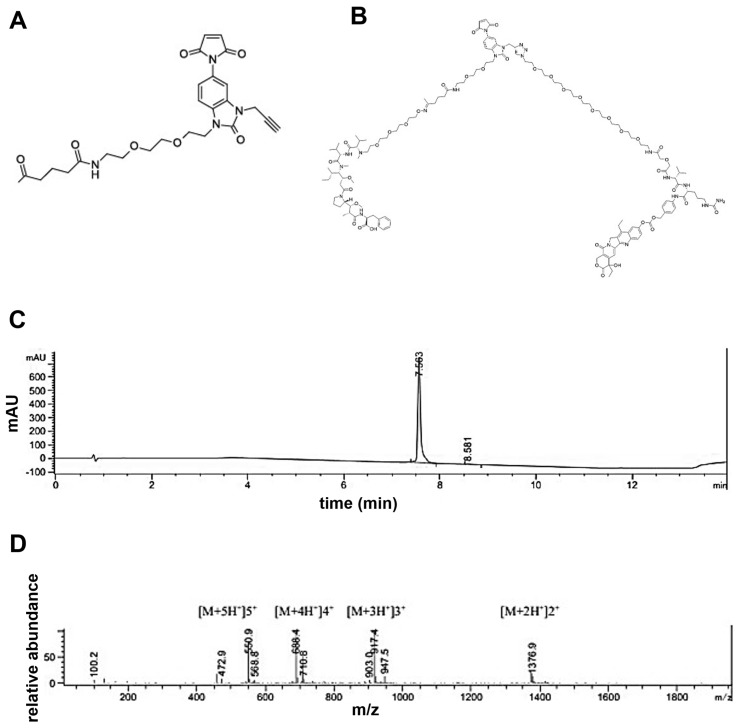
A tri-functional linker was developed to attach two payloads to an antibody. **(A**) Structure of our novel linker functionalized with maleimide, propargyl, and ketone groups. (**B**) Structure of linker with PEG4-MMAF and PEG8-Valine-Citruline-PABC-SN38 payloads attached. (**C**) Purity of the trifunctional linker with the conjugated MMAF and SN38 payloads was confirmed using reversed phase chromatography. The chromatogram has absorbance at 220 nm in milli-Absorbance Units (mAU) in the Y axis and retention time in minutes (min) in the X axis. (**D**) Mass spectrum of the peak representing the linker with the conjugated MMAF and SN38. The spectrogram has relative abundance in the Y-axis and mass to charge ration (m/z) in the X-axis. Peaks are annotated with protonated theoretical mass and ionization states.

A novel tri-functional linker was synthesized (Figure 1A) and then coupled to MMAF and SN38 to generate a dual-payload molecule (Figure 1B). The structure of the linker with the dual payload was confirmed using HPLC (Figure 1C) and mass spectrometry (Figure 1D). The HPLC data showed a single peak indicating a pure product. The mass spectrum demonstrated the expected molecular weight of the intact dual-payload molecule.

### 3.2. ADCs with Dual and Single Payloads Were Successfully Conjugated

**Figure 2:**
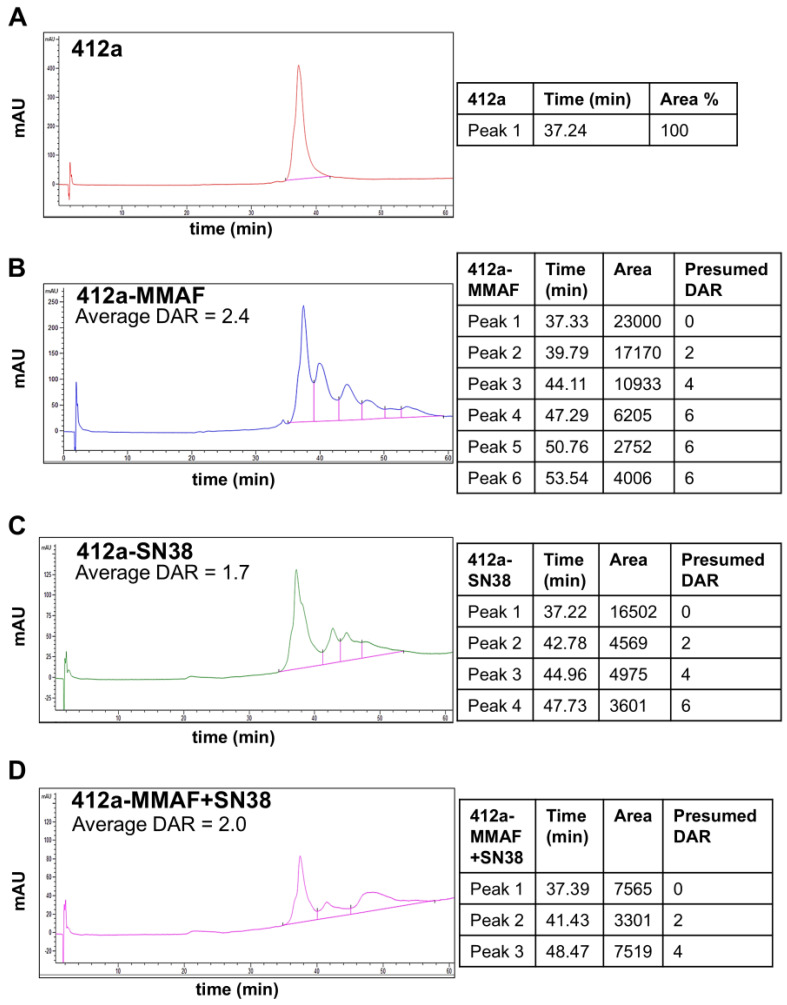
Hydrophobic interaction chromatography of ADCs. Hydrophobic interaction chromatography (HIC) was performed on (**A**) 412a, (**B**) 412a-MMAF, (**C**) 412a-SN38, and (**D**) 412a-MMAF+SN38 to confirm conjugation. The chromatograms show absorbance at 214 nm in milli-Absorbance Units (mAU) on the Y-axis and elution time in minutes (min) on the X-axis. A baseline was drawn from the bottom of the leading edge of the first peak to the bottom of the tailing edge of the latest peak. The areas under each peak were calculated from the baseline. The presumed DAR assignment of each peak was theoretical for the cysteine conjugations. The HIC analyses confirmed the conjugation of 412a with single and dual payloads.

The tri-functional linker with MMAF and SN38 was conjugated to a bi-specific antibody against EGFR and cMET (412a) using maleimide conjugation to produce a targeting dual-payload ADC (412a-MMAF+SN38). To generate single-payload controls, MC-MMAF and CL2A-SN38 were conjugated to 412a. The ADC conjugations were confirmed using hydrophobic interaction chromatography. Unconjugated 412a eluted at 37.2–37.4 min, and conjugates at increasing drug-to-antibody ratios eluted at later peaks corresponding to their increasing hydrophobicity (Figure 2).

### 3.3. 412a-MMAF+SN38 Was More Potent and Efficacious In Vitro than the Single-Payload ADCs

**Figure 3:**
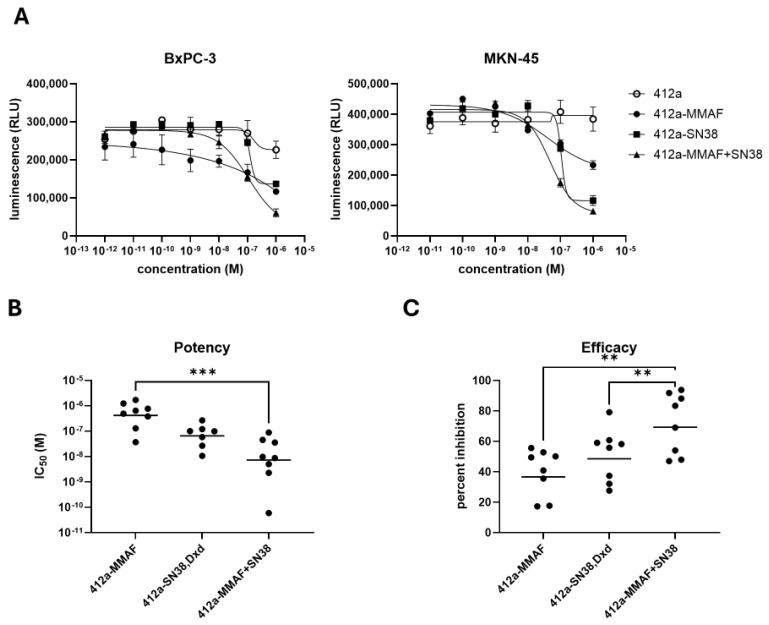
A 412a ADC with dual MMAF and SN38 payloads improved potency and efficacy over single-payload 412a ADCs. A panel of 8 cell lines consisting of gastric, pancreatic, small cell lung, and triple negative breast cancer cell lines were treated with a molar (M) dilution series of 412a-MMAF, 412a-SN38 or 412a-druxtecan (Dxd), 412a-MMAF+SN38 dual-payload ADC, and unconjugated 412a for 72 h. Cell viability at each concentration of ADC was determined using a luminescent readout of ATP levels in relative light units (RLU). (**A**) Data are shown from two representative cancer cell lines, BxPC-3 and MKN45 cells. Curves are fitted using a nonlinear regression. (**B**) Half-maximal inhibitory concentration (IC_50_) values were generated from cell viability curves to determine potency of each test article. Each data point represented a different cell line. Groups without 8 data points had cell lines that were not responsive enough to the ADC to generate an IC_50_ value. *** = *p* < 0.001 (**C**) Efficacy values for each test article in 8 cell lines were determined using the formula (untreated cells—ADC treated cells)/untreated cells ×100. ** = *p* < 0.01.

To assess how well the dual-payload molecule inhibited tumor cell growth, a variety of gastric, pancreatic, small cell lung, and triple negative breast cancer cell lines were treated with 412a alone, 412a-MMAF, 412a-SN38, and 412a-MMAF+SN38. The ATP levels were assessed as readouts for cell viability using a luminescence assay. The growth inhibition curves from the BxPC-3 and MKN-45 cells showed more complete growth inhibition after treatment with the 412a-MMAF+SN38 molecule when compared to 412a-MMAF or 412a-SN38 single-payload ADCs (Figure 3A). The potency of the 412a-topoisomerase inhibitor (SN38 or Dxd), 412a-MMAF, and 412a-MMAF+SN38 was determined by using nonlinear regression modeling of the growth inhibition curves to find the IC_50_ values of each drug (Figure 3B). The dual-payload 412a-MMAF+SN38 was significantly more potent than 412a-MMAF and more potent than 412a-SN38.

The efficacy was also evaluated by calculating the percent reduction in cell numbers between the untreated wells and the maximum dose of drug (Figure 3C). The 412a-MMAF+SN38 treatment had a significantly larger percent reduction when compared to the 412a-MMAF or 412a-SN38 treatment meaning that fewer viable cells were left after dual-payload treatment compared to single-payload treatments. Taken together, dual-payload 412a-MMAF+SN38 was more potent and efficacious than single-payload 412a ADCs.

### 3.4. Nondividing Cell Models to Assess Dual-Payload ADC Efficacy in Cell States Less Susceptible to ADC-Mediated Killing

**Figure 4:**
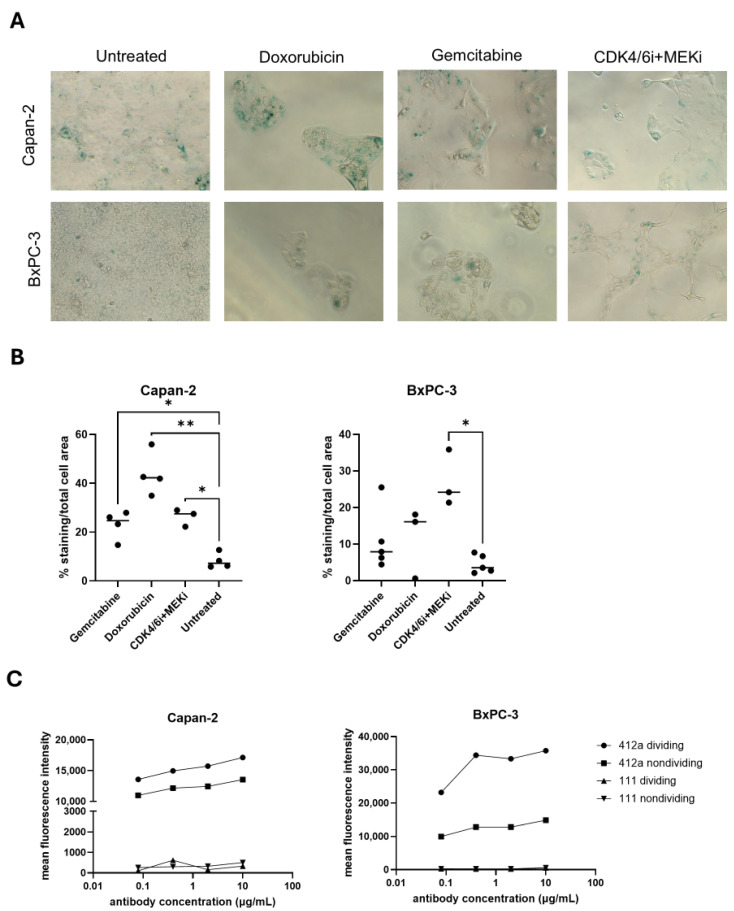
Pancreatic cancer cell lines formed senescent populations in response to chemotherapy and targeted therapy. BxPC-3 and Capan-2 cells were untreated or treated with 100 nM gemcitabine, 100 nM doxorubicin, or a combination of 1 μM ribociclib and 5 μM binimetinib (CDK4/6i+MEKi) for 6–7 days to establish nondividing cell models. (**A**) Cells were stained for SA-β-gal expression. Representative 20x images show expression in blue. (**B**) SA-β-gal expression was quantified from BxPC-3 and Capan-2 cells. Each data point is an average of two wells with four images taken per well. * = *p* < 0.05, ** = *p* < 0.01 (**C**) Capan-2 cells were treated with doxorubicin and BxPC-3 cells were treated with CDK4/6i+MEKi to become nondividing. Both dividing and nondividing Capan-2 and BxPC-3 cells were incubated with 412a or 111 (nonbinding control) antibody to assess antibody binding in both populations. An Alexa Fluor-647 conjugated secondary antibody against 412a and 111 was used for detection. Mean fluorescence intensity of the Alexa Fluor-647 antibody at multiple concentrations of 412a and 111 in micrograms/milliliter (mg/mL) is displayed.

We developed resistance models based on senescent cell phenotypes. We treated BxPC-3 and Capan-2 cells with a variety of drugs that induced senescence. After 7 days of treatment, we stained for SA-β-gal function and imaged cells to assess expression (Figure 4A) [30]. Doxorubicin and gemcitabine were assessed at concentrations ranging from 10^−10^ to 10^−7^ M (Supplementary Figure S2) with 10^−7^ M doxorubicin and gemcitabine producing the highest levels of SA-β-gal expression in Capan-2 cells. CDK4/6i+MEKi were added at a set combination of 1 µM CDK4/6i and 5 µM MEKi. After 6–7 days, images of Capan-2 and BxPC-3 cells treated with doxorubicin, gemcitabine, or CDK4/6i+MEKi showed that the strongest increase in SA-β-gal was found in Capan-2 cells treated with doxorubicin and BxPC-3 cells treated with CDK4/6i+MEKi. The staining profiles from the four separate images were quantified in Figure 4B.

Surface protein presentation can be altered when cells enter senescence. To determine if 412a was able to bind to the senescent or nondividing Capan-2 and BxPC-3 cells, Capan-2 cells were treated for one week with doxorubicin and BxPC-3 cells were treated for a week with CDK4/6i+MEKi. After a week, cells were incubated with an escalating dose of 412a or a nonbinding bispecific antibody (111) from 0.1 µg/mL to 10 µg/mL. Figure 4C shows mean fluorescence intensity of the secondary antibody at each dose of 412a or 111 in dividing and nondividing Capan-2 and BxPC3 cells. As expected, increasing doses of 412a resulted in higher levels of binding, while increasing doses of 111 did not change binding. In both BxPC-3 and Capan-2 models, less 412a bound to nondividing cells indicating a downregulation of EGFR and/or cMET on the cell surface although binding was still well above background levels. Although a slight reduction in binding occurred, 412a targeted nondividing Capan-2 and BxPC3 cells.

### 3.5. 412a-MMAF+SN38 Maintained Superior Efficacy over Single-Payload ADCs in Dividing and Nondividing Cells

**Figure 5:**
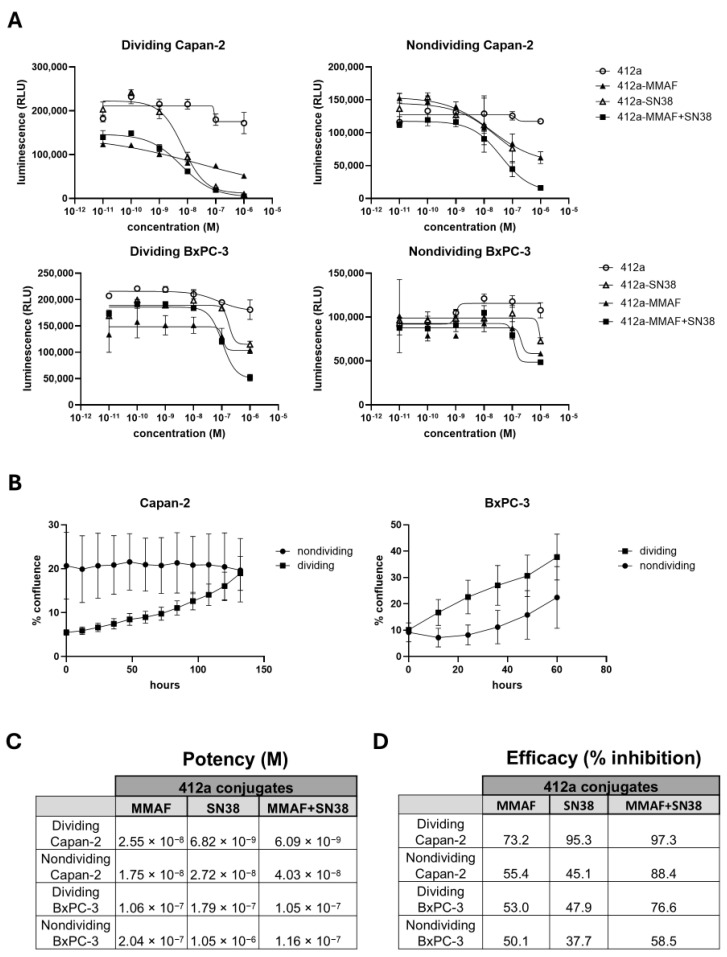
The dual-payload 412a-MMAF+SN38 ADC retains superior potency and efficacy in dividing and nondividing cells. BxPC-3 was treated with ribociclib and binimetinib (CDK4/6i+MEKi) and Capan-2 cells were treated with doxorubicin to establish nondividing cell models. Dividing and nondividing cells were treated with a range of concentrations of 412a-MMAF, 412a-SN38, 412a-MMAF+SN38, or unconjugated 412a. (**A**) Cell viability was determined using a luminescent readout of ATP levels in relative light units (RLU). Curves showing cell viability at each ADC concentration are displayed. (**B**) Dividing and nondividing cells were placed in an Incucyte after treatment with ADCs. Percent (%) confluence of untreated dividing and nondividing Capan-2 and BxPC-3 cells over time are graphed. (**C**) Cell viability graphs were used to determine potency by calculating molar (M) IC_50_ values for ADCs in each cell type. (**D**) Efficacy was determined by comparing the readout from the highest concentration of each ADC to the untreated group using the formula: (untreated cells—ADC treated cells)/untreated cells × 100.

The potency and efficacy of the dual-payload 412a-MMAF+SN38 ADC was compared to the single-payload ADCs in cells that were more resistant to ADC killing. First, we generated nondividing Capan-2 and BxPC-3 cells as outlined previously. Dividing and nondividing BxPC3 cells were treated with the ADCs for 3 days and, because of their slow growth kinetics, dividing and nondividing Capan-2 cells were treated with the ADCs for 6 days (Supplementary Figure S3). Due to differences in cell viability over time with the senescence-inducing drugs, the ADC was added to doxorubicin-containing media for the Capan-2 cells while CDK4/6i+MEKi was removed from BxPC-3 cells prior to ADC treatment. At the end of the treatment, the viable cells were assessed using a luminescent readout of ATP levels. As seen previously, 412a-MMAF+SN38 treatment led to greater percent killing than that of the single-payload 412a-MMAF or 412a-SN38 ADCs (Figure 5A,C). The 412a-MMAF+SN38 showed a higher percent inhibition of the nondividing Capan-2 cells than that of the single-payload ADCs. The nondividing BxPC-3 cells were less inhibited by ADC than the nondividing Capan-2 cells. However, dividing and nondividing BxPC-3 cells still had higher percent inhibition with 412a-MMAF+SN38 treatment compared to single-payload treatment. The lack of ADC-mediated killing in the nondividing BxPC-3 cells could come from having a shorter 3 day exposure or because they lost more EGFR/cMET binding capacity (Figure 4C). Regardless, 412a-MMAF+SN38 still had superior percent inhibition when compared to the single-payload controls in both the dividing and nondividing models.

When dividing and nondividing BxPC-3 and Capan-2 cells were treated with ADC, the Incucyte assessed confluence every 12 h. The confluence of the untreated dividing and nondividing cells assured that the nondividers were not replicating during the course of ADC treatment (Figure 5B). While the confluence of the Capan-2 cells did not change during the experiment, the BxPC-3 cells did start to regrow, albeit with a slower growth rate than that of the dividing cells. This was most likely due to the selective pressure of drug being removed from the BxPC-3 cells when the ADC was added. Therefore, while the Capan-2 model was truly nondividing, the BxPC-3 model more closely resembled differences in the slow growing versus the rapidly dividing tumor cells.

### 3.6. 412a-MMAF+SN38 Inhibited Tumor Growth More Effectively than Single-Payload ADCs at Lower Concentrations

A single dose of 412a-MMAF+SN38 was administered to non-tumor-bearing Balb/c Nude mice to determine the concentration of ADC in serum over time. Using a Human IgG ELISA, 412a was detected in serum 240 h after administration of 412a-MMAF+SN38 with a half-life of 88.6 h (Supplementary Figure S4). Using these pharmacokinetic data, we set a dosing schedule of twice per week for two weeks so the drug would not fall far below 50% concentration in serum before the subsequent dose.

**Figure 6:**
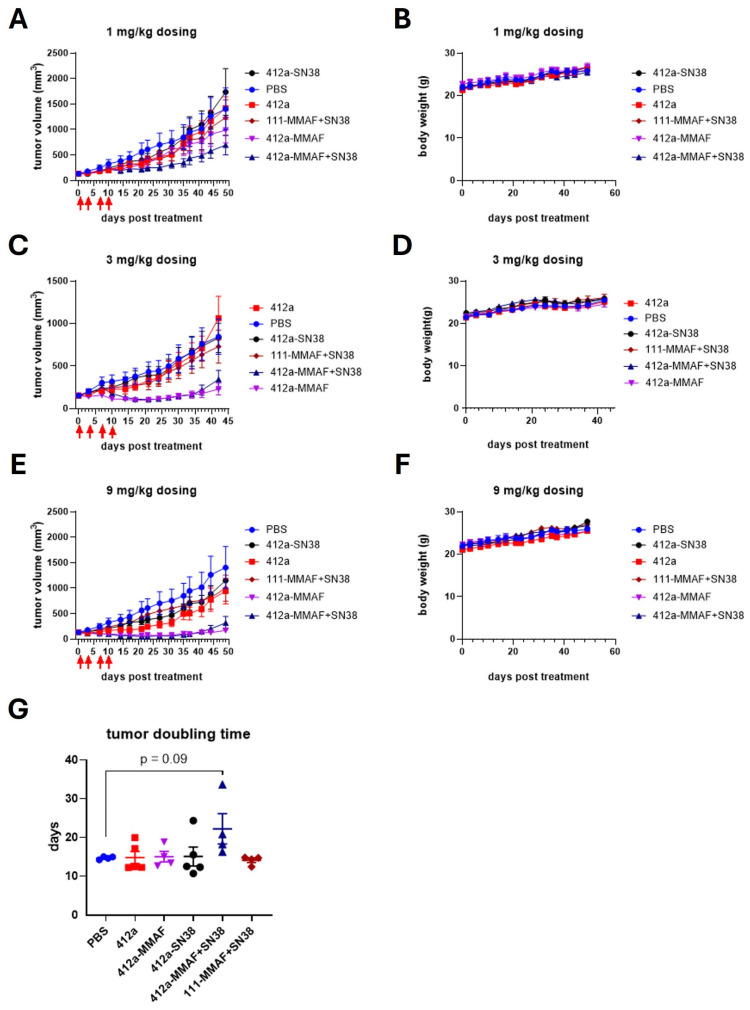
412a-MMAF+SN38 more potently inhibits tumor growth at low doses compared to single-payload ADCs. BxPC-3 cells were implanted subcutaneously into the flank of Balb/c Nude mice (*n* = 4–5). When tumor volume reached approximately 150 mm cubed (mm^3^), 412a-SN38, 412a-MMAF, 412a-MMAF+SN38, and 111-MMAF+SN38 (nonbinder) ADCs were administered along with 412a only and PBS controls twice weekly for a total of four doses. The red arrows indicate specific dosing days. Tracings show average tumor growth for each treatment condition over time at 1 milligram per kilogram (mg/kg) (**A**), 3 mg/kg (**C**), or 9 mg/kg (**E**) doses. Body weight after 1 mg/kg (**B**), 3 mg/kg (**D**), or 9 mg/kg (**F**) doses were also recorded. (**G**) Average tumor doubling time for each animal in the 1 mg/kg dosing group is shown. All data points represent the mean value ± standard error of the mean.

To determine if 412a-MMAF+SN38 was more effective at inhibiting tumor growth in vivo than 412a-MMAF or 412a-SN38, BxPC-3 cells were injected subcutaneously into the flank of Balb/c Nude mice. Once the tumors reached 100–200 mm^3^, 412a-MMAF, 412a-SN38, 412a-MMAF+SN38 were administered via intraperitoneal injection along with 412a, PBS, and the nonbinding dual-payload ADC 111-MMAF+SN38 as controls. The tumor growth was assessed in response to 1 mg/kg (Figure 6A), 3 mg/kg (Figure 6C), and 9 mg/kg (Figure 6E) doses of ADC. Animals were dosed twice per week for two weeks as indicated by the red arrows on the graphs showing tumor growth. At 3 mg/kg and 9 mg/kg doses, 412a-MMAF and 412a-MMAF+SN38 similarly stopped tumor growth for around 40 days. At the 1 mg/kg dose, 412a-MMAF+SN38 more effectively inhibited tumor growth compared to 412a-MMAF although tumors growth was only stopped for around 25 days before steadily regrowing. To quantify the changes in tumor growth at 1 mg/kg, tumor size was modeled as a function of time to determine the rate at which the tumors doubled in size (Figure 6G). Doubling time of the tumors treated with 412a-MMAF+SN38 was notably longer than all other groups. Surprisingly, 412a-SN38 was not effective at any dose.

The body weight of the mice was also recorded throughout the experiment as a preliminary indicator of tolerability (Figure 6B,D,F). None of the test articles led to decreases in body weight at any dose, suggesting that 412a-MMAF+SN38 did not cause any obvious tolerability issues when compared to single-payload ADCs at similar or higher DAR. However, additional toxicology experiments could be performed in the future to more thoroughly compare 412a-MMAF+SN38 to single-payload ADCs. Overall, 412a-MMAF+SN38 was shown to be better at inhibiting tumor growth at lower doses compared to 412a-MMAF or 412a-SN38.

## 4. Discussion

We have constructed a novel bi-specific (412a), dual-payload ADC by attaching an antibody targeting EGFR and cMET that are overexpressed in many tumor types. We also selected MMAF and SN38 payloads to use in our proprietary tri-functional linker because they were clinically validated payloads to which many tumor types were sensitive and have distinct mechanisms of action. Both SN38 and Dxd were evaluated in vitro as single-payload ADCs with similar potency and efficacy. The non-diffusible payload MMAF was also chosen instead of the diffusible MMAE payload to complement diffusible SN38, even though MMAE has been used more often in ADCs. Pairing diffusible and non-diffusible payloads could leverage the bystander killing of diffusible payloads with the reliability of non-diffusible payloads, which are less affected by drug efflux pumps.

The 412a-MMAF+SN38 conjugates were monodisperse and maintained their activity over 2 months at 4 °C. 412a-MMAF+SN38 was significantly more potent and efficacious when compared to 412a-MMAF and 412a-topoisomerase inhibitor single-payload ADCs in a panel of 8 cancer cell lines consisting of gastric, pancreatic, small cell lung, and triple negative breast cancers. Both 412a-SN38 and 412a-Dxd were tested as topoisomerase inhibitors and had similar trends in potency and efficacy across cell lines. These cancer cell lines were also specifically chosen as they represented tumors that were difficult to treat and had relatively few therapeutic options. Additionally, 412a-MMAF+SN38 was tested in nondividing and slow cycling tumor cell models and continued to show better efficacy when compared to 412a-MMAF or 412a-SN38 ADCs. In the in vivo BxPC-3 model, 412a-MMAF+SN38 also inhibited tumor growth for a longer time period at the lowest 1 mg/kg dose. This demonstrated how the dual-payload ADC was more effective at inhibiting tumor growth and could be a beneficial alternative to the single-target, single-payload ADCs in the market. While the results are promising in cell line-derived xenograft models, further verification in patient-derived xenograft models would better assess clinical relevance [31].

The Capan-2 and BxPC-3 models were used to depict resistant cell states that could lead to poor payload efficacy in nondividing populations. We characterized SA-β-gal activity as a qualitative measure of the nondividing cell state [32]. Independent of senescence, the upregulation of SA-β-gal could also be associated with other cell states [33], which was important to acknowledge when considering the BxPC-3 model. When treated with CDK4/6i+MEKi for more than a week, most of the cells initiated cell death even though they expressed SA-β-gal and had senescent-like morphologies. However, upon removal of CDK4/6i+MEKi, the cells eventually started to regrow, indicating the presence of a more heterogeneous population. Such heterogeneous models could better represent patient tumors since the true distribution of senescent cells and rapidly dividing cells could not be well defined in patients. Regardless of the differences, both of the nondividing models demonstrated reduced responses to our ADCs when compared to rapidly growing cells. Therefore, they were still appropriate models for ADC resistance. Target downregulation and resistance to payload were likely to be critical resistance mechanisms limiting therapeutic efficacy of approved ADCs. While 412a target downregulation was seen in our models of nondividing cells, future experiments could assess how the dual-payload ADC could be re-engineered to compensate for resistance to each individual target.

An additional caveat to our work was that the ADC drug antibody ratio (DAR) distribution was different between the dual and single-payload conjugates. The 412a-MMAF ADC was the easiest to conjugate with an approximate DAR of 2.4 with ADC species ranging with DAR 2–6, while 412a-SN38 and 412a-MMAF+SN38 were conjugated closer to an average DAR of 2. Although the DAR could be important when considering drug potency, the 412a-MMAF+SN38 with a DAR of 2 had better anti-tumor growth inhibition activity than the single payload 412a-MMAF ADC with a DAR of 2.4 at low doses. While reports have shown little difference between ADCs with DAR 2 and DAR 6 when assessing in vitro and in vivo efficacy [34], this could impact the profiles of in vivo tumor growth inhibition. Although ADCs with high DAR are generally more potent, they have also been shown to exhibit faster clearance and result in a reduced therapeutic index [34]. Exploring the impact of dual-payload DAR on ADC clearance and in vivo stability could be explored in future work.

While the 412a-SN38 ADC was effective against BxPC-3 cells in vitro, it was less effective against BxPC-3 tumors in vivo. Additional experiments could determine the cause of this discrepancy. Clinically effective SN38-bearing ADCs generally have a higher DAR than both auristatin-bearing ADCs and our 412a-SN38 ADC. We therefore hypothesized that the 412a-SN38 ADC showed no efficacy in vivo because the DAR was too low to inhibit tumor growth. While the linker used was clinically validated, we did not directly examine the in vivo stability of the ADC. Thus, it was also possible that the in vivo PK was insufficient to deliver SN38 in an effective manner. In vivo stability, PK/PD, and toxicology studies are goals for future work.

## 5. Conclusions

Overall, our novel dual-target, dual-payload ADC showed promise compared to single-payload ADCs. With our tri-functional linker, we could also easily replace the antibodies and payloads. We demonstrated how such design can be a platform to generate ADCs to overcome barriers such as acquired resistance in a range of tumor types to better serve patients with cancers of unmet need.

## Data Availability

The original contributions presented in this study are included in the article/Supplementary Material. Further inquiries can be directed to the corresponding author.

## Supplementary Materials

The following supporting information can be downloaded at: https://www.mdpi.com/article/10.3390/pharmaceutics17080967/s1, Figure S1: Synthetic route of linker–dual payload linker construction; Figure S2: Senescent cell percentages increase with doxorubicin and gemcitabine concentration; Figure S3: Schematic of the timeline for treating dividing and nondividing cells with antibodies and ADC; Figure S4: 412a-MMAF+SN38 is present in serum 10 days post-injection.

## Abbreviations

The following abbreviations are used in this manuscript:

ADC	Antibody–drug conjugate
MMAF	Monomethyl auristatin F
FDA	U.S. Food and Drug Administration
EGFR	Epidermal growth factor receptor
cMET	Mesenchymal–epithelial transition factor
FBS	Fetal bovine serum
DMSO	Dimethyl sulfoxide
CDK4/6i	Cyclin-dependent kinase 4/6 inhibitor
MEKi	Mitogen-activated protein kinase inhibitor
SA-β-gal	Senescence-associated beta-galactosidase
RLU	Relative light units
BSA	Bovine serum albumin
FSC	Forward scatter
SSC	Side scatter
HPLC	High performance liquid chromatography
HIC	Hydrophobic interaction chromatography
mAU	Milli-absorbance units
IC_50_	Half-maximal inhibitory concentration
PBS	Phosphate-buffered saline
LC-MS	Liquid chromatography-mass spectrometry
Dxd	Deruxtecan
