# Supplementary material for: A Dual-Payload Bispecific ADC Improved Potency and Efficacy over Single-Payload Bispecific ADCs
Source: Pharmaceutics. 2025 Jul 25;17(8):967. doi: 10.3390/pharmaceutics17080967 (PMC12389611; doi:10.3390/pharmaceutics17080967)
Supplement: Supplementary file 1 [file pharmaceutics-17-00967-s001.zip › pharmaceutics-3703737 supplementary figure legends.pdf]

SUPPLEMENTARY FIGURE LEGENDS

Supplementary Figure 1: Synthetic route of linker–dual payload linker construction. Compound **1** (5 mg, 1.0eq) was added to a solution of compound **2** (10.85 mg, 1.2 eq) and m-phenylenediamine catalyst (1.3 mg, 1.2 eq) in 2.0 ml (PBS:DMSO, 10:1, V:V). The reaction was stirred for 12 h at room temperature. The reaction solution was detected by LC-MS. The crude material was purified by RP-HPLC to obtain compound **3** as light yellow powder, (ES+) m/z (relative intensity) ([M+H]+:1415.8). Compound **3** (5.0 mg, 1.0 eq) was added to a solution of CuSO4 (0.1 mg, 0.15 eq), sodium ascorbate (0.21 mg, 0.3 eq) and Compound **4** (5.65mg, 1.2eq) in 3 mL (1-Butanol: H2O, 2:1, V:V). The reaction was stirred for 24 h at room temperature. The reaction solution was detected by LC-MS. The reaction solution was filtered by 0.45um filter membrane and then purified by RP-HPLC to obtain compound **5** as light yellow powder, (ES+) m/z (relative intensity) ([M+2H]2+:1376.9).

Supplementary Figure 2: Senescent cell percentages increase with doxorubicin and gemcitabine concentration. BxPC-3 and Capan-2 cells were treated with a range of molar (M) concentrations of gemcitabine and doxorubicin to determine the optimal concentration for establishing a nondividing cell model. Percent SA-β-gal staining expression is shown for each concentration, which was calculated using the following formula: SA-β-gal area/total cell area x 100.

Supplementary Figure 3: Schematic of the timeline for treating dividing and nondividing cells with antibodies and ADC.

Supplementary Figure 4: 412a-MMAF+SN38 is present in serum 10 days post-injection. A single 3 mg/kg dose of 412a-MMAF+SN38 was administered to non-tumor-bearing female Balb/c nude mice via intraperitoneal injection. Serum samples were collected from two groups of mice (n = 3 per group) at alternating time points of 1, 24, 72, and 168 hours post-injection and 6, 48, 120, and 240 hours post-injection. Serum concentrations of 412a-MMAF+SN38 were quantified using a commercially available Human IgG Precoated ELISA Kit. A) Mean and standard error of all points were shown. A nonlinear regression was performed to model loss of the drug in serum over time. B) Pharmacokinetic modelling was performed using ADAPT 5 software to determine half-life and Cmax values.
