# Supplementary material for: A Dual-Payload Bispecific ADC Improved Potency and Efficacy over Single-Payload Bispecific ADCs
Source: Pharmaceutics. 2025 Jul 25;17(8):967. doi: 10.3390/pharmaceutics17080967 (PMC12389611; doi:10.3390/pharmaceutics17080967)
Supplement: Supplementary file 1 [file pharmaceutics-17-00967-s001.zip › pharmaceutics-3703737 supplementary figures.pdf]

Supplementary Figure 1

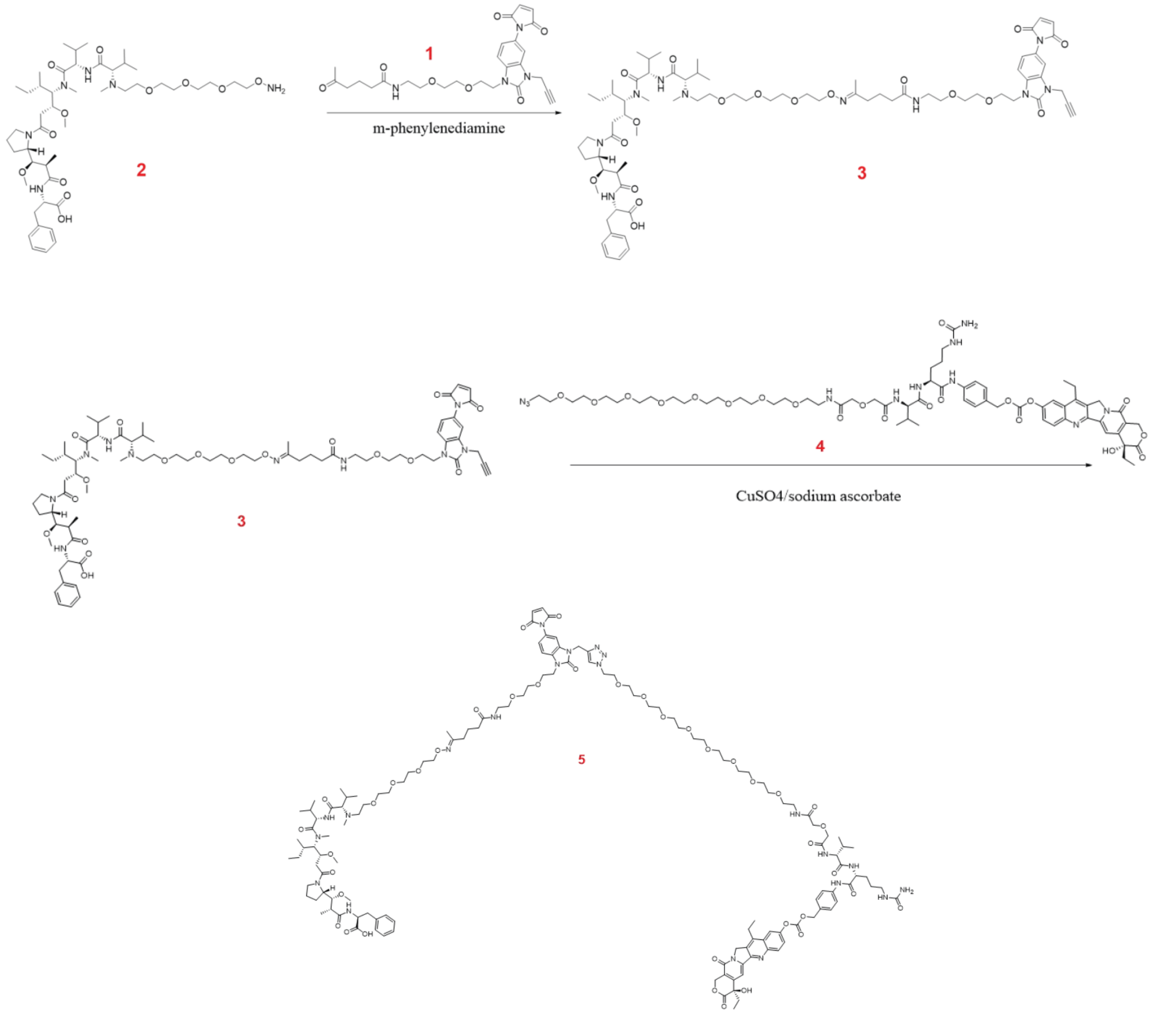

Supplementary Figure 2

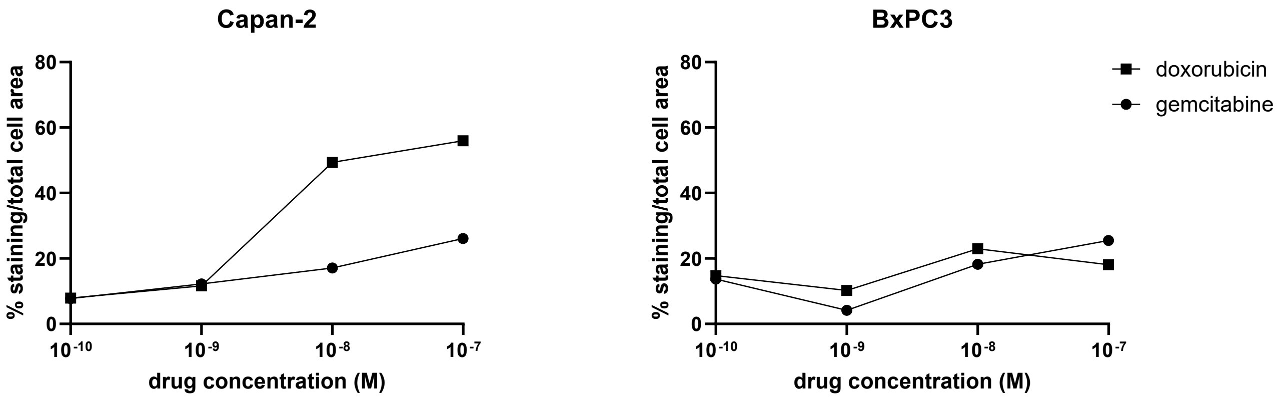

Supplementary Figure 3

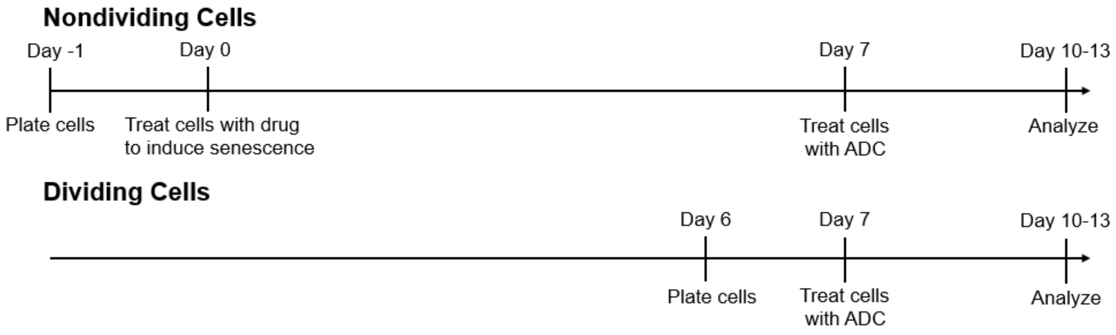

Supplementary Figure 4

A

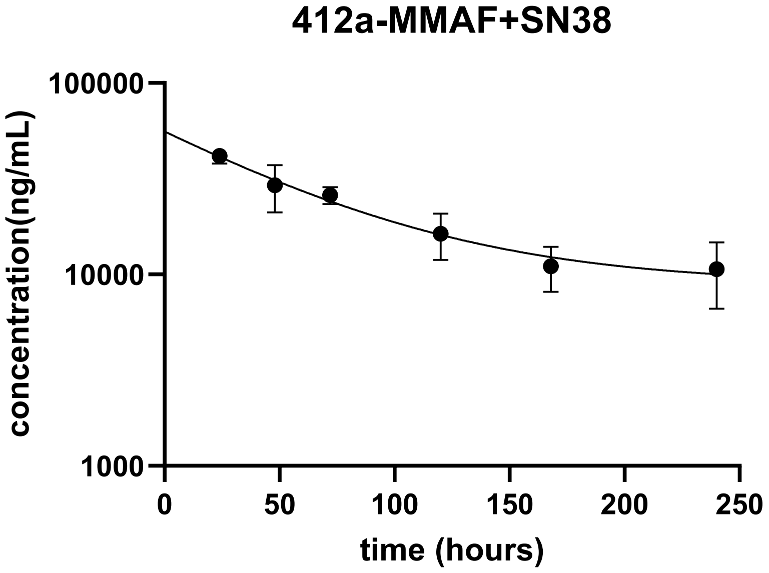

B

412-MMAF+SN38 PK	
**Cmax**	54452.9 ng/mL
**Half-life**	88.6 hours
